# Reviewer Acknowledgments

**DOI:** 10.13023/jah.0501.09

**Published:** 2023-04-01

**Authors:** Randy Wykoff, Rachel E. Dixon

**Affiliations:** East Tennessee State University, wykoff@etsu.edu; Journal of Appalachian Health, managingeditor.jah@gmail.com

**Keywords:** Appalachia, peer review, thank-you

## Abstract

As we reach our fifth year, we are particularly thankful for the contributions of our peer reviewers. The knowledge, expertise, and guidance offered by the people listed below have ensured that we can continue to share timely research to effect health and well-being across Appalachia.

As we mark, with this issue’s publication, the beginning of our fifth year, we want to reflect on the enormous—and too often thankless—contribution of our network of peer reviewers. The people listed below have volunteered their time, energy, and attention to ensure that this Journal remains a credible, up-to-date outlet for knowledge on Appalachian health and well-being. We owe an enormous debt of gratitude to these content experts who have cheerfully shepherded our articles into stronger, more relevant pieces with their constructive reviews. Theirs is the work that upholds one of the most important aspects of academic pursuit: the peer review process.

Alongside our reviewers, we thank the members of our Editorial Board and Advisory Board, East Tennessee State University (our new managing institution), the University of Kentucky (our publisher), and the institutions who have partnered with us. Together, we have navigated a year of considerable change and tremendous growth. We mourn those who have left us and appreciate the energy brought by new joiners. Though we offer it here, our heartfelt “thanks” can never be enough to convey the appreciation we hold for this community of people, all of whom have given generously of their time and minds in the service of Appalachia.

Abram Brummettt

Christiaan G. Abildso

Lacey Andrews

Thomas Arcury

Elizabeth Ayangunna

Md. Tofial Azam

Casey P. Balio

Rebecca Battista

Ruchi Bhandari

Kristine Bowers

Billy Brooks

Kimberly A. Carney

W. Jay Christian

Jamison Conley

Wayne Coombs

Matthew Courser

Cory E. Cronin

Alan M. Ducatman

Cecily Fassler

Judith Feinberg

Brian W. Flynn

Arthur L. Frank

Tim J. Hilbert

Monique Hill

Sydeena E. Isaacs

Kingsley A. Kalu

Raihan Khan

Brenna Kirk

Jennifer R. Larson

Jonathon Leider

Sue Lin

Herb Linn

Kristin Lunz Trujillo

Hadii M. Mamudu

Malissa McAlister

Randy P. McCombie

Denise Moreno Ramírez

Todd P. Newman

Bethesda O’Connell

Robert Pack

Margo Riggs

Christine Schalkoff

Ashlyn Nicole Schwartz

David A. Shoham

Adams Sibley

Kostas Skordas

Rick Slaven

Michael Smith

Laura Swan

Jessica Thompson

Jennifer Tyson

Robin C. Vanderpool

Amy Wahlquist

Lisa Washburn

Sallie J. Weaver

Rachel L. Wright

Daniel Yoder

Jennie L. Yoost

